# Aldose Reductase Involvement in EMT: Emerging Insights and Current Proposed Molecular Mechanisms

**DOI:** 10.3390/biology14101422

**Published:** 2025-10-16

**Authors:** Gemma Sardelli, Francesca Felice, Rossella Mosca, Martina Avanatti, Roberta Moschini

**Affiliations:** Biochemistry Unit, Department of Biology, University of Pisa, 56123 Pisa, Italy; gemma.sardelli@phd.unipi.it (G.S.); francesca.felice@unipi.it (F.F.); r.mosca@studenti.unipi.it (R.M.); m.avanatti@studenti.unipi.it (M.A.)

**Keywords:** aldose reductase, AKR1B1, EMT, fibrosis, metastasis

## Abstract

**Simple Summary:**

Aldose reductase is a protein involved in several important processes in the body, such as sugar metabolism, reducing harmful molecules, and managing inflammation. While its role in protecting cells from damage and processing sugars is well understood, other functions remain unclear. Recent research has linked aldose reductase to a process called the epithelial–mesenchymal transition (EMT). EMT is a natural process important for development and wound healing but can also contribute to diseases like cancer (helping cancer spread) and fibrosis (organ scarring). Studies suggest that aldose reductase actively promotes EMT in both cancerous and non-cancerous conditions. For example, it may help cancer cells spread in breast cancer and worsen lung fibrosis in non-cancer cases. However, scientists still don’t fully understand how aldose reductase triggers EMT. This review brings together findings from different studies, exploring potential mechanisms behind this link. It also examines how blocking aldose reductase might affect EMT, offering insights into how the enzyme could be targeted in future treatments. This overview highlights what is currently known about aldose reductase and EMT while identifying unanswered questions to guide further research.

**Abstract:**

Aldose reductase (AKR1B1) is a member of the aldo-keto reductase (AKR) family and plays a variety of roles in many metabolic and physiological processes. Although its function in the polyol pathway and defense against reactive carbonyl species is well-documented, many of aldose reductase’s roles remain poorly understood. Recently, accumulating evidence has suggested a strong correlation between aldose reductase expression and/or activity and the epithelial-to-mesenchymal transition (EMT), a process fundamental to both physiological conditions (e.g., embryonic development and wound healing) and pathological states (such as fibrosis and metastasis). Specifically, aldose reductase appears to be a potent promoter of EMT in both tumor and non-tumor contexts, although the molecular mechanisms through which it drives EMT remain unclear. This review aims to summarize the growing body of studies highlighting the association between AKR1B1 and EMT, as well as to analyze the molecular mechanisms proposed by various authors. Finally, the main findings on EMT responses following AKR1B1 inhibition will be discussed, providing a comprehensive overview of current knowledge and identifying the critical gaps that must be addressed to fully elucidate the role of aldose reductase in this process.

## 1. Introduction

The epithelial-to-mesenchymal transition (EMT) is a highly coordinated biological process whereby epithelial cells lose their polarity and intercellular adhesion, acquiring mesenchymal traits that confer enhanced motility and invasiveness [1]. While EMT is essential for physiological processes such as embryogenesis, wound healing, and tissue remodeling, it also serves as a key driver of pathological conditions, including fibrosis, cancer progression and metastasis. Depending on the signals received by a cell within its tissue microenvironment and the activated cell-specific gene regulatory network, this program drives cells through a series of intermediate phenotypic states along the epithelial–mesenchymal axis [2]. As a rapidly evolving field, new molecular mechanisms and regulators are continually being identified, expanding the already intricate network of pathways that govern EMT. In this view, increasing evidence supports a strong association between aldose reductase (AKR1B1) and EMT, in both tumorigenic and non-tumorigenic settings, primarily through its roles in redox homeostasis, inflammatory signaling pathways, and metabolic reprogramming.

AKR1B1 is a cytosolic enzyme traditionally studied in the context of diabetes and metabolic disease due to its role in the polyol pathway, where it catalyzes the reduction of glucose to sorbitol [3]. Beyond this classical metabolic function, AKR1B1 has emerged as a significant player in oxidative stress responses, inflammation, and tumor development [4,5]. Given the growing number of studies across diverse pathological conditions that converge on a tight association between AKR1B1 and EMT, there is a need to consolidate current knowledge on this non-canonical function of AKR1B1. This review aims to provide a comprehensive overview of the existing evidence linking AKR1B1 to EMT, and to summarize the major molecular mechanisms proposed to underlie this association across different biological contexts.

### 1.1. EMT: Definition and Biological Contexts

EMT refers to a complete reprogramming of gene expression that converts epithelial cells into cells with mesenchymal properties, occurring physiologically during embryonic development, tissue regeneration, and wound healing, and pathologically in conditions such as organ fibrosis and metastasis [6]. Epithelial cells typically exhibit apical-basal polarity, where the apical and basolateral surfaces have distinct appearances and functions. These cells are closely associated with one another through specialized cell–cell junctions, such as tight junctions, adherens junctions, and desmosomes [7]. Tight junctions are complexes located near the apical surface and serve as membrane fusions that provide intercellular sealing and maintain cell compartmentalization. Occludin and claudin are important components of the intercellular tight junction strands, while the cytoplasmic components Zonula Occludens (ZO)-1, -2, -3 and p120 are essential for their connection with actin filaments, thus contributing to the strength and integrity of tight junctions [8]. Adjacently to tight junctions and facing the basolateral side, adherens junctions link epithelial cells to the cytoskeletal microfilaments. The transmembrane adhesion protein E-cadherin, which characterizes adherens junctions, engages in homotypic interactions via its ectodomain, whilst the cytoplasmic domain of E-cadherin binds to β-catenin, which interacts with α-catenin, anchoring the complex to the actin cytoskeleton [9].

EMT is often initiated in response to certain specific biochemical and mechanical stimuli, most notably transforming growth factor (TGF)-β, which signals through a heteromeric complex of two types (I and II) transmembrane serine-threonine kinase receptors [10]. Upon ligand binding, the type II receptor kinase phosphorylates the type I receptor, leading to the activation of the downstream intracellular signal typically distinguished in Smad-dependent or Smad-independent [11]. Briefly, following the TGF-β binding, Smad-dependent signaling initiates with proteins Smad2 and 3 which are recruited to the receptor via the adaptor protein SARA. After phosphorylation by the receptor kinase activity, the activated Smad2/3 is released from the receptor complex and subsequently forms a heterodimeric complex with Smad4 and accumulates in the nucleus to regulate gene transcription. Alternatively, TGF-β binding may trigger Smad-independent signaling responses, such as Erk/MAP kinases, Rho GTPases and the PI3 kinase/Akt pathways [11]. These pathways converge to activate master regulators of EMT, belonging to three main families, such as SNAIL (including Snail and Slug), helix-loop-helix (HLH) (with Twist1 and 2), and ZEB (ZEB1 and 2) [10]. Upon activation, these transcription factors in turn repress epithelial marker gene expression and concomitantly activate mesenchymal gene expression. Consequently, one of the first events in EMT is the disassembly of tight and adherens junctions, leading to the loss of cell polarity and the initiation of cytoskeletal reorganization [12]. Notably, the loss of E-cadherin is considered a hallmark of EMT, accompanied by the upregulation of N-cadherin, a protein commonly found in non-epithelial tissues, particularly endothelial cells. The acquisition of N-cadherin is critical for enabling mesenchymal cells to interact with non-epithelial tissues, especially with blood vessel cells, thereby facilitating their entry into the bloodstream and subsequent dissemination [13].

Concomitantly, cells undergoing EMT acquire a mesenchymal identity, characterized by the expression of mesenchymal cytoskeletal proteins such as vimentin, and the increased deposition of extracellular matrix (ECM) components, including collagen and fibronectin. These ECM components stimulate integrin signaling and promote the formation of focal adhesion complexes, which are essential for cell migration [2]. Additionally, EMT is associated with the upregulation of several matrix metalloproteases (MMPs), which facilitate the remodeling of basement membranes and allow cells to detach and migrate, also activating latent growth factors, including further activation of TGF-β, thus creating a self-reinforcing EMT loop [14].

### 1.2. Aldose Reductase: Functions and Biological Implications

Aldose Reductase (Aldo-keto reductase family 1 member B1, AKR1B1) is a cytosolic enzyme belonging to the aldo-keto reductase (AKR) superfamily, which catalyzes the NADPH-dependent reduction in a wide variety of carbonyl-containing substrates [15]. Due to its high structural flexibility, AKR1B1 exhibits broad substrate specificity and functional versatility. Structurally, the enzyme features a central (α/β) 8-barrel, where β-strands and α-helices are connected by three highly flexible loops (loops A, B, and C) located at the back of the barrel, which are critical in determining substrate specificity. This structural arrangement allows aldose reductase to function as a wide-specificity catalyst [16].

AKR1B1 is best known for initiating the polyol pathway, an alternative glucose metabolic route. Under normoglycemic conditions, only ~3% of intracellular glucose is metabolized through this pathway, but during hyperglycemia, its activity can increase up to 30% of total glucose metabolism, making it one of the most important collateral pathways contributing to glucose toxicity [17].

The polyol pathway comprises two enzymatic steps: first, AKR1B1 reduces glucose to sorbitol using NADPH as a cofactor. Second, sorbitol is oxidized to fructose by sorbitol dehydrogenase (SDH) in an NAD^+^-dependent reaction [18]. Sorbitol, being a highly hydrophilic polyol, cannot easily diffuse across cell membranes, leading to intracellular accumulation. This causes osmotic stress due to water influx. The fructose produced can be phosphorylated to fructose-6-phosphate and re-enter glycolysis or feed the hexosamine biosynthetic pathway, particularly in cells expressing hexokinase isoforms I–III, which, although less efficient than hexokinase IV, are capable of fructose phosphorylation [19]. The resultant fructose metabolism contributes to both glycative and oxidative stress. Hyperactivation of this pathway has been strongly implicated in diabetic complications [20,21], and pharmacological inhibition of AKR1B1 has been shown to prevent, delay, or in some cases even reverse tissue damage associated with chronic hyperglycemia [18].

Beyond its metabolic role, AKR1B1 also contributes to redox homeostasis. The enzyme can detoxify reactive aldehydes generated during lipid peroxidation, including 4-hydroxynonenal (4-HNE) and its glutathione-conjugated form GS-HNE [22,23,24]. Through this activity, AKR1B1 acts as an antioxidant enzyme, and its expression is transcriptionally regulated by Nrf2, a master regulator of oxidative stress responses [25]. Moreover, AKR1B1-mediated reduction of GS-HNE to GS-DHN has been shown to activate key inflammatory pathways such as NF-κB and AP-1, implicating the enzyme in inflammation-driven pathology [26,27]. AKR1B1 also participates in prostaglandin synthesis. In normal conditions, phospholipids are converted into arachidonic acid in a reaction catalyzed by phospholipases A2 (PLA2G) enzyme and subsequently converted to prostaglandin H2 (PGH2) with the via cyclooxygenase 1 (COX1) and COX2 [28]. AKR1B1 then converts PGH2 to prostaglandin F2-α (PGF2A), consuming NADPH [29].

Emerging evidence also suggests a role for AKR1B1 in cancer biology, where it may influence pathways regulating inflammation, apoptosis, chemoresistance, and cell cycle progression. These effects are mediated through complex interactions with signaling molecules, transcription factors, and miRNAs which are not yet fully elucidated, but collectively point to AKR1B1 as a significant contributor to cancer progression and aggressiveness [5]. Consistently, as recently reviewed by Nagini et al., AKR1B1 expression has been found to be upregulated in several cancers and associated with shortened patient survival [30].

Interestingly, a growing number of studies across diverse pathological conditions converge on a tight association between AKR1B1 and EMT, although there is a clear need to consolidate current knowledge on this non-canonical function. In the following sections a comprehensive overview of the existing evidence linking AKR1B1 to EMT will be provided, reporting the major proposed molecular mechanisms to mediate this association across different cellular contexts.

## 2. Dysregulated AKR1B1 Expression and EMT

Dysregulation of AKR1B1 has emerged as a key factor promoting EMT across a wide spectrum of pathological conditions. EMT, a cellular program essential during development and wound healing, becomes pathologically activated in fibrosis, chronic inflammation, and cancer, contributing to disease progression by enabling cellular migration, invasion, and phenotypic plasticity. Increasing evidence supports a central role for AKR1B1 in mediating EMT through context-specific molecular pathways. A distinction can be made between AKR1B1-driven EMT occurring in tumor settings and that which takes place in non-tumor contexts.

### 2.1. Aldose Reductase in Non-Tumoral EMT

In non-tumoral diseases, the role of AKR1B1 in EMT has been primarily investigated in the context of chronic tissue remodeling, fibrosis, and organ dysfunction—conditions frequently associated with hyperglycemia, in which the enzyme is overexpressed and hyperactivated. For instance, in diabetic nephropathy—a progressive kidney disease driven by sustained hyperglycemia and characterized by excessive ECM deposition, mesangial expansion, thickening of the glomerular and tubular basement membranes, and tubulo-interstitial fibrosis [31]—AKR1B1 has been identified as a promoter of EMT and fibrogenesis in tubular epithelial cells. This occurs through the downregulation of the miR-200a-3p/141-3p axis, which in turn leads to the activation of TGF-β1/2 and ZEB1/2 signaling pathways [32].

Another diabetic complication involving EMT is diabetic cataract, characterized by lens opacification and visual impairment [33]. In this context, EMT plays a crucial role because it is responsible for the loss of cell polarity, cell-to-cell contact, and epithelial characteristics of lens epithelial cells (LECs). Transformed cells may then proliferate and migrate to the visual axis, resulting in opacification and reduced visual acuity [34]. Wu and collaborators demonstrated in two subsequent investigations that, in diabetic lenses, AKR1B1 overexpression inhibits AMPK activity, leading to acetylation and inactivation of superoxide dismutase 2 (SOD2). This process ultimately exacerbates oxidative stress and promotes EMT via RAGE signaling [35,36]. A form of secondary cataract arising from aberrant proliferation and trans-differentiation of residual LECs following cataract surgery is the posterior capsule opacification (PCO) [37], mainly initiated by TGF-β which becomes elevated in the eye as part of the surgical wound response [38]. TGF-β binds to its receptor on the surface of LECs, inducing them to undergo EMT. As a result, LECs begin migrating toward the posterior aspect of the lens capsular bag, secrete ECM proteins, and promote fibrosis and wrinkling of the capsular bag. During PCO, AKR1B1 has been shown to facilitate EMT in two independent studies [39,40], in both cases involved in TGF- β signaling.

Independently on hyperglycemic conditions, another pathological setting characterized by EMT-mediated tissue remodeling is pulmonary fibrosis, in which injury to alveolar epithelial cells and fibroblast proliferation lead to fibroblast activation and abnormal ECM deposition, ultimately compromising respiratory function [41]. Li et al. reported EMT-associated upregulation of AKR1B1 using both in vitro and in vivo models of pulmonary fibrosis, although the underlying mechanisms remain incompletely understood [42]. Notably, the in vivo findings mirrored the in vitro observations, supporting a consistent profibrotic role of AKR1B1 across experimental systems. Exposure of cultured pulmonary fibroblasts to TGF-β1 for 24 h significantly increased both mRNA and protein levels of AKR1B1, as well as expression of fibrotic markers α-SMA and collagen I. Moreover, silencing AKR1B1 expression via siRNA or pharmacological inhibition reversed the TGF-β1-induced upregulation of these fibrotic markers. Zhang and coworkers further demonstrated that AKR1B1-induced EMT plays a critical role in radiation-induced pulmonary fibrosis (RIPF), a progressive lung condition that arises as a late adverse effect of thoracic radiotherapy [43]. Using AKR1B1-knockout (KO) and wild-type (WT) mice exposed to thoracic radiation, the authors showed that AKR1B1 deficiency significantly attenuated fibrotic responses. This was evidenced by reduced expression of collagen I and MMP2, decreased mesenchymal markers (α-SMA, vimentin), and increased E-cadherin levels in KO mice relative to WT controls. Complementary, in vitro experiments using murine lung epithelial (MLE-12) cells revealed that radiation-induced EMT, cell migration, and upregulation of both AKR1B1 and the EMT transcription factor Twist1 were significantly diminished by pharmacological inhibition or siRNA-mediated knockdown of AKR1B1 [43]. These data demonstrate a strong concordance between cellular and animal models, reinforcing the causal role of AKR1B1 in radiation-induced EMT and fibrosis.

Additionally, Yadav and collaborators demonstrated that AKR1B1 plays a pivotal role in allergen-induced airway remodeling in asthma by promoting EMT via a PI3K/AKT/GSK3β-dependent signaling pathway, although the exact molecular mechanisms were not fully delineated [44]. Using an ovalbumin-induced mouse model of lung inflammation, as well as in vitro systems employing human small airway epithelial cells (SAECs) and murine lung fibroblasts, the authors observed that TGF-β1 treatment led to reduced expression of epithelial markers (E-cadherin, occludin) and increased expression of mesenchymal markers (vimentin, MMP-2, α-SMA, fibronectin). These changes were significantly attenuated by AKR1B1 inhibition using Fidarestat which markedly suppressed the activation of AKT, GSK3β, and PAK kinases—thereby preventing GSK3β inactivation and subsequent Snail-mediated E-cadherin repression, but without altering SMAD2/3 phosphorylation. In vivo, Fidarestat-treated asthmatic mice also showed reduced phosphorylation of PI3K and GSK3β, further supporting a role for AKR1B1 in mediating TGF-β1-induced EMT and airway remodeling through non-canonical signaling pathways [44]. The studies reported above strongly sustain the role of AKR1B1 role as molecular modulators of signaling pathways responsible for EMT induction both in vitro and in vivo models. Also, the parallel outcomes obtained in cell-based assays and in the ovalbumin-induced mouse model emphasize the translational relevance of AKR1B1-dependent EMT signaling.

### 2.2. Aldose Reductase in Tumoral EMT

In tumoral settings, EMT plays a pivotal role in driving metastasis. Metastasis is a multistep process wherein tumor cells detach from the primary tumor lesion, invade the surrounding stroma, intravasate into the microvasculature, survive circulatory shear stress, extravasate into distant tissues, and ultimately establish secondary tumors [45]. EMT facilitates nearly every stage of this cascade by promoting the loss of intercellular adhesion, cytoskeletal remodeling, and acquisition of migratory and invasive capabilities. An increasing number of studies have highlighted a prominent role for AKR1B1 in EMT-driven metastasis across various solid tumor types.

In basal-like breast cancer, an aggressive triple-negative subtype of mammary cancer, AKR1B1 is transcriptionally activated by Twist2 and sustains a positive feedback loop involving NF-κB activation via PGF2A, thereby reinforcing EMT and enhancing tumor invasiveness. These findings were corroborated in xenograft models, where AKR1B1 overexpression enhanced metastatic dissemination, confirming its relevance beyond cell-based system [46]. Similarly, in lung, breast and ovarian cancers, AKR1B1 is indirectly upregulated by ZEB1, and in turn promotes autocrine TGF-β signaling through the polyol pathway, supporting EMT and metastatic dissemination [47]. Consistent findings in lung cancer and glioblastoma indicate that AKR1B1 has been shown to promote EMT via activation of the RhoA–ROCK2 pathway, thereby enhancing migratory and invasive behavior [48].

Additional evidence from gastric cancer has shown that fructose generated via AKR1B1 activity activates a novel KHK-A/YWHAH/SLUG signaling axis that represses E-cadherin and induces EMT, a key mechanism underlying cancer cell detachment and invasiveness [49]. More recently, Yang and colleagues demonstrated that AKR1B1 interacts with STAT3 and indirectly support EMT, enhancing tumor cell survival under oxidative stress [50].

In hepatocellular carcinoma, the most common form of primary liver cancer, AKR1B1 has been shown to bind the kinase domain of AKT1, leading to activation of the AKT/mTOR pathway. This signaling cascade promotes metabolic reprogramming (Warburg effect and lactate production), inflammation, and EMT, ultimately driving tumor progression [51].

Finally, in colorectal cancer, AKR1B1 contributes to EMT through multiple mechanisms. One study identified its role in NF-κB activation via the formation of GS–DHN adducts, which leads to upregulation of genes associated with migration and invasion [52]. Additional studies have linked AKR1B1 to increased cell motility [53] and poor clinical prognosis, potentially through activation of the p70S6K signaling pathway [54], although the precise molecular mechanisms remain less characterized.

Taken together, these findings underscore the pathological significance of AKR1B1 in promoting EMT in both neoplastic and non-neoplastic conditions. Whether mediating fibrotic remodeling or facilitating metastatic dissemination, AKR1B1 functions as a central mediator linking metabolic and inflammatory stress to cellular plasticity and tissue invasion. This broad involvement highlights AKR1B1 as a promising therapeutic target for EMT-driven diseases and for counteracting tumor progression and spread. For this purpose, a clear understanding of its molecular function in the aforementioned contexts is essential. In the following section, we summarize the proposed molecular mechanisms to provide an integrated framework that may guide future research and therapeutic development.

## 3. Proposed Molecular Mechanisms

The functional contribution of AKR1B1 to EMT appears to involve both enzymatic (catalytic) mechanisms and non-enzymatic mechanisms independent of its catalytic activity, with their relative contribution varying according to the biological context. AKR1B1 enzymatic activities can be further distinguished into glucose-dependent (linked to the polyol pathway) and glucose-independent (involving alternative carbonylic substrates). Collectively, these activities converge on transcriptional and metabolic pathways that promote the acquisition of mesenchymal traits. This dual role positions AKR1B1 as a distinctive regulator of cellular plasticity, integrating extracellular stress signals with intracellular metabolic reprogramming and inflammatory cascades. While the precise mechanisms remain incompletely defined, several candidate pathways have been proposed.

### 3.1. Glucose-Dependent Enzymatic Mechanisms

The role of AKR1B1 in promoting EMT often involves its enzymatic function within the polyol pathway, resulting in alteration of osmotic and redox balance which activate downstream signaling cascades.

Wei et al. showed that in diabetic nephropathy, AKR1B1 drives renal EMT and fibrosis by repressing the protective miR-200a/141 axis and impairing Nrf2 antioxidant signaling. Using mesangial cells and diabetic mouse models, they observed that high glucose, channeled into the polyol pathway through AKR1B1, rapidly and markedly suppressed miR-200a-3p/141-3p expression. Consequently, the expression of their target Keap1, TGF-β1/2, and ZEB1/2 results increased, ultimately promoting fibrogenesis and EMT [32]. Interestingly, they observed that pharmacological inhibition of AKR1B1 effectively diminished high-glucose-induced suppression of miR-200a-3p, hampering renal fibrogenesis and EMT. This suggests that, under hyperglycemic stress, its glucose reductase activity is essential for fibrosis and diabetic kidney disease [32].

Chang et al. demonstrated that AKR1B1 promotes EMT in LECs through non-canonical TGF-β signaling. In transgenic mice, AKR1B1 overexpression increased TGF-β2 and mesenchymal markers (α-SMA, fibronectin, Snail) while reducing epithelial markers (E-cadherin, Foxe3, Pax6). AKR1B1 inhibition reversed most of these changes, restoring E-cadherin and reducing fibronectin/α-SMA levels. Notably, EMT induction occurred independently of SMAD2 activation but was associated with enhanced ERK1/2 phosphorylation, which was suppressed by AKR1B1 inhibition. These findings indicate that AKR1B1-driven polyol pathway activity promotes redox imbalance and EMT via MAPK/ERK signaling, contributing to fibrotic remodeling in posterior capsular opacification [40].

Two subsequent investigations, conducted by Wu and colleagues, highlighted the role of AKR1B1 in mediating EMT in diabetic cataracts through oxidative stress–related mechanisms [35,36]. Firstly, the researchers proposed that AKR1B1 overexpression in diabetic lenses contributes to AMPK activation and consequent SOD2 acetylation (resulting in a less effective defense against superoxide anion). This leads to redox imbalance, elevated ROS and AGE production, promoting EMT via RAGE and ROS signaling and contributing to fibrotic lens changes and cataract progression [35]. Building on this, the authors then demonstrated that the treatment with D3T—a naturally occurring antioxidant compound from cruciferous vegetables and AKR1B1 inhibitor—significantly reversed these effects, reducing SOD2 acetylation, restoring active AMPK levels, and suppressing EMT transcription factors [36].

Beyond diabetes, emerging evidence is convincing that polyol pathway is indispensable for cancer growth and survival, and its end-product fructose is an important fuel for lung cancer survival and therapy resistance [55]. Using various cancer cell lines and NSCLC patient samples, Schwab et al. demonstrated that the polyol pathway, through AKR1B1 activity, connects glucose metabolism EMT and cancer progression [47]. On the basis of their observations, researchers proposed that excess glucose fuels EMT via AKR1B1-mediated polyol pathway activity and autocrine TGF-β stimulation. Additionally, this leads to the activation of ZEB1 transcription factor, which indirectly positively regulates AKR1B1 expression, thus sustaining a positive feedback loop and EMT [47].

Similarly, Kang et al. demonstrated that in gastric cancer, the hyperglycemia-induced increase in intracellular fructose—from polyol pathway—drives cytoskeletal rearrangement, E-cadherin downregulation, and increased cell migration. These changes were functionally dependent on KHK-A kinase. Researchers proposed that, under high glucose and fructose conditions, KHK-A underwent dimer dissociation and nuclear translocation (via interaction with proteins LRRC59 and KPNB1), where it phosphorylated YWHAH at serine 25. This phosphorylation promoted the recruitment of the SLUG transcriptional repressor to the E-cadherin promoter, thereby repressing its expression and inducing EMT. Inhibition of AKR1B1 or SORD, or competition with L-fructose, successfully reversed these EMT phenotypes both in vitro and in vivo, including suppression of metastasis in xenografted diabetic mice [49].

In parallel, Zhao et al. confirmed in both lung cancer and glioblastoma models that cancer cells actively convert glucose into fructose via the polyol pathway, and this endogenous fructose strongly supports tumor proliferation and migration. Using ^13^C-glucose tracing, they showed that 11 out of 12 cancer cell lines convert glucose into labeled fructose. AKR1B1 genetic deletion or pharmacological inhibition reduced fructose production, leading to impaired proliferation and migration in A549 (lung) and U87 (glioblastoma) cells. Notably, exogenous fructose supplementation restored cell growth and mobility, emphasizing the importance of fructose as a driver of malignancy. AKR1B1 knockout cells also showed reduced metastasis in vivo, along with altered EMT marker expression and marked downregulation of RhoA and ROCK2, components of a signaling pathway associated with cytoskeletal dynamics and motility [48].

### 3.2. Glucose-Independent Enzymatic Mechanisms

Alongside its role in polyol pathway, AKR1B1 has a well-recognized role in reactive aldehyde detoxification and prostaglandin production and, as such, key enzyme in inflammatory states. In this context, Tammali et al. demonstrated that AKR1B1 plays a central role in colon cancer metastasis by promoting NF-κB–dependent expression of pro-metastatic genes. In colon cancer cell lines (HT29 and KM20), stimulation with growth factors such as EGF and FGF increased cancer cell proliferation, migration, invasion, and adhesion to endothelial cells. These effects resulted significantly attenuated by blocking AKR1B1 activity or expression. In vivo experiments further confirmed that systemic AKR1B1 inhibition led to a ~65% reduction in liver metastases. Mechanistically, AKR1B1 activity was associated with increased activation of the NF-κB p65 subunit and upregulation of key pro-metastatic factors such as MMP2, cyclin D1, and CD34. These findings highlight AKR1B1’s enzymatic role—likely through metabolism of lipid aldehydes—in triggering NF-κB signaling and enhancing metastatic potential [52]. Building on this mechanistic framework, Wu et al. identified a positive feedback loop between AKR1B1, NF-κB, and Twist2 in basal-like breast cancer that sustains EMT and CSC-like traits. They showed that the transcription factor Twist2 directly binds the AKR1B1 promoter, inducing its expression. AKR1B1, in turn, increases PGF2A production, which activates NF-κB signaling. Activated NF-κB (particularly RelA) then enhances Twist2 transcription, creating a self-reinforcing circuit. This loop downregulates E-cadherin while promoting mesenchymal markers such as vimentin and N-cadherin, driving EMT [46].

### 3.3. Non-Catalytic Mechanisms

Beyond its well-established enzymatic functions in glucose and reactive aldehyde metabolism, AKR1B1 also exerts non-enzymatic (catalysis-independent) effects by acting as a protein scaffold or signaling modulator. In this view, AKR1B1 directly interacts with key signaling proteins, thereby influencing intracellular pathways without requiring substrate consumption. Such interactions modulate the EMT process and are particularly relevant in cancer progression and fibrotic disease contexts.

In hepatocellular carcinoma, AKR1B1 promotes tumorigenesis by interacting directly with key components of the AKT/mTOR signaling pathway [51]. Mechanistically, AKR1B1 physically interacted with AKT1, as demonstrated by co-immunoprecipitation, leading to upregulation of p-AKT1, AKT2, p-FOXO1, mTOR, HIF-1α, PKM2, and SREBP. These effects resulted in enhanced lactate production and lactate dehydrogenase (LDH) activity, indicative of metabolic reprogramming toward the Warburg effect. Importantly, this metabolic shift and signaling activation were abolished by AKT1 knockdown or inhibition, confirming its essential contribution. In vivo, liver-specific overexpression of AKR1B1 enhanced AKT/mTOR signaling and promoted hepatocarcinoma development in diethylnitrosamine (DEN)-induced mouse hepatic tumor models [51].

In gastric cancer, AKR1B1 plays a pro-tumorigenic role through modulation of ferroptosis via interaction with STAT3 and p-STAT3 [50]. Functionally, AKR1B1 interacted directly with STAT3 and p-STAT3, leading to enhanced SLC7A11 transcription. SLC7A11 mediates cystine uptake, needed for glutathione de novo synthesis and crucial for the suppression of ferroptosis. Consistently, the knockdown of AKR1B1 significantly decreased SLC7A11 expression, whilst the overexpression of SLC7A11 counteracted ferroptosis and restored cell proliferation and invasion, confirming the AKR1B1–STAT3–SLC7A11 axis as a key pathway in gastric cancer progression, independently of AKR1B1’s enzymatic activity, reinforcing its role as a signaling scaffold in cancer [50]. Of note, the AKR1B1-STAT3 interaction leading to upregulation of SLC7A11 and enhanced cystine transport, has also been demonstrated in lung cancer, where it contributes to drug resistance [56].

In parallel with the oncologic context, AKR1B1 promotes EMT in LECs through direct interaction with SMAD2, a central effector of TGF-β signaling [39]. Treatment of human and murine LECs with AKR1B1 siRNA or inhibitor significantly reduced TGF-β2–induced migration, SMAD2/3 phosphorylation, MMP-2/MMP-9 activity, and expression of mesenchymal markers (α-SMA, vimentin). Immunoprecipitation and proximity ligation assays (PLA) confirmed a direct interaction between AKR1B1 and SMAD2, which was dependent on NADPH binding but independent of its catalytic activity. This interaction enhances SMAD2 phosphorylation and facilitates its nuclear translocation, thereby amplifying EMT-associated transcription [39].

**Figure 1:**
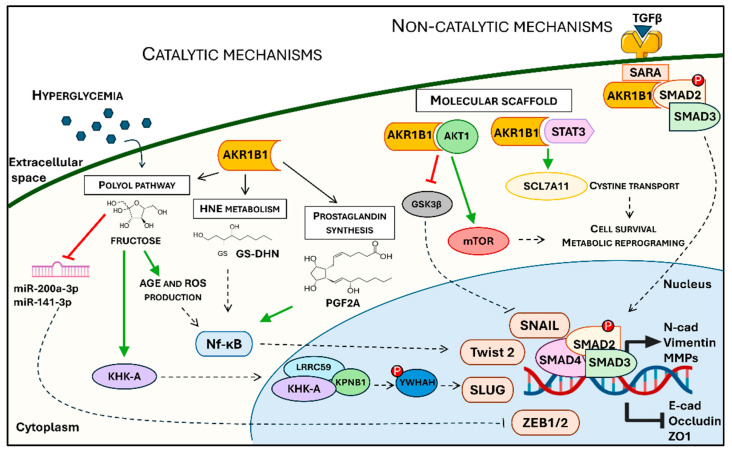
Schematic representation of proposed mechanisms mediated by AKR1B1 in EMT progression. As a key cytosolic aldo-keto reductase enzyme, AKR1B1 participates in several catalytic pathways including the polyol pathway, 4-hydroxy-2-nonenal (HNE) metabolism, and prostaglandin synthesis, which contribute to advanced glycation end-product (AGEs) and reactive oxygen species (ROS) generation, NF-κB activation, and the fructose-induced downstream pathway via KHK-A. In parallel, non-catalytic roles of AKR1B1 involve acting as a molecular scaffold for AKT1 and STAT3, promoting downstream signaling through mTOR and cystine transport (via SLC7A11), and interacting with SMAD2 in TGF-β–mediated SMAD2/3 activation. These signaling cascades converge on EMT transcription factors (SNAIL, SLUG, Twist2, ZEB1/2), leading to repression of epithelial markers and induction of mesenchymal markers (N-cadherin, vimentin, MMPs), thereby supporting cell survival and metabolic reprogramming.

Overall, AKR1B1 can drive EMT through enzymatic mechanisms, by catalyzing carbonyl reduction, or through non-enzymatic mechanisms, by interacting with other proteins and signaling complexes, and these modes of action might not be mutually exclusive. The proposed molecular mechanisms detailed in this paragraph are graphically summarized in Figure 1.

## 4. Clinic Implications and Therapeutic Opportunities

As stated in previous paragraphs, AKR1B1’s enzymatic activity appears crucial in promoting EMT in numerous circumstances. Thus, its pharmacological inhibition using AKR1B1 inhibitors may offer a promising strategy to target EMT-related pathways across various diseases and tumor types, underscoring the novel therapeutic potential of AKR1B1. Being the first enzyme in the polyol pathway, AKR1B1 is a critical pharmacological target in efforts to prevent diabetic complications. Numerous Aldose Reductase Inhibitors (ARIs) were identified—belonging to three representative structural classes, namely carboxylic acid derivatives, cyclic imides [57], and phenolic derivatives [58]—but very few are in clinical trials or approved for clinical use [18].

Epalrestat, a carboxylic acid derivative, is the only approved and commercially available ARI to date, largely used in Japan for the targeted treatment of diabetic complications [59]. Epalrestat has been reported to consistently counteract AKR1B1-driven EMT and metastatic processes across different tumoral contexts in preclinical studies. In basal-like breast cancer, Epalrestat disrupted the Twist2–AKR1B1–NF-κB positive feedback loop, restored E-cadherin expression, reduced PGF2A production, and markedly suppressed cell migration, invasion, and lung metastasis in vivo [46]. In gastric cancer under hyperglycemic conditions, it attenuated fructose-induced cytoskeletal rearrangement, cell migration, and E-cadherin repression by interfering with the polyol pathway–driven activation of the KHK-A–YWHAH–SLUG axis [49]. More recently, in lung and glioblastoma models, Epalrestat suppressed glucose-to-fructose conversion through the polyol pathway, reducing cancer cell proliferation, migration and metastasis, effects that could be rescued by exogenous fructose supplementation [48].

Tolrestat is another ARI of carboxylic acid derivative group. Zhang and colleagues reported that AKR1B1 inhibition with Tolrestat significantly counteracts radiation-driven EMT and fibrogenesis [43]. In irradiated lung epithelial MLE-12 cells, Tolrestat treatment prevented the loss of E-cadherin, reduced the upregulation of mesenchymal markers (α-SMA, vimentin), and suppressed collagen I and MMP2 expression. Importantly, Tolrestat also attenuated radiation-enhanced cell migration and inhibited the induction of Twist1, a key EMT transcription factor [43].

Within cyclic imides group of inhibitors, the most utilized ARIs are Fidarestat and Sorbinil, both have been shown to suppress AKR1B1-driven EMT processes in both cancer and non-tumor contexts. In allergen-induced airway remodeling, Fidarestat reversed TGF-β1–induced EMT in human airway epithelial cells and mouse lung fibroblasts, restoring E-cadherin and occludin expression while preventing upregulation of vimentin, α-SMA, fibronectin, and MMP2 by interfering with the PI3K/AKT/GSK3β signaling, thereby blocking Snail activation and EMT progression [44]. In colon cancer, both Fidarestat and Sorbinil inhibited EGF/FGF-induced growth, migration, and invasion of HT29 and KM20 colon cancer cells, reduced adhesion of tumor cells to growth factor–stimulated endothelial cells and significantly decreased liver metastasis in vivo [52]. Additionally, Chang and coworkers demonstrated that Sorbinil treatment opposed AKR1B1-induced EMT by maintaining E-cadherin expression while reducing α-SMA, fibronectin, and vimentin levels [39,40].

Lastly, the inhibitory effects of dietary polyphenols on AKR1B1—including flavonoids, catechins, stilbenes, chalcones, and anthocyanidins—have attracted considerable research interest [60]. However, there is currently no direct evidence demonstrating that these compounds can counteract EMT through AKR1B1 inhibition, and this research gap warrants further investigation.

## 5. Conclusions

In this review we report a comprehensive overview of the current knowledge supporting AKR1B1’s role in the EMT process. AKR1B1 has emerged as a key regulator of the EMT, a process that underlies the progression and complications of a wide range of pathologies, including fibrosis, diabetes, and cancer. Evidence across different models indicates that AKR1B1 promotes EMT through multiple, context- and cell-type-dependent molecular mechanisms. These can be broadly divided into catalytic mechanisms—either glucose-dependent, via the polyol pathway, or glucose-independent, involving other carbonyl substrates—and non-catalytic mechanisms, such as protein–protein interactions and structural support. Together, these functions highlight AKR1B1 as a central node within a complex regulatory network whose full scope remains to be elucidated.

Importantly, inhibition of AKR1B1 has consistently proven effective in preclinical studies for attenuating EMT, thereby reducing fibrotic progression and metastatic dissemination. This positions AKR1B1 as a promising therapeutic target for diseases in which EMT acts as a pathological driver, with broad implications for oncology, fibrotic disorders, and metabolic disease complications. Despite strong mechanistic evidence, no AKR1B1 inhibitor has achieved global clinical approval, mainly due to off-target effects, low bioavailability, limited efficacy, and the enzyme’s dual physiological and pathological roles. Early candidates, such as Sorbinil and Tolrestat, were discontinued because of adverse effects and poor pharmacokinetic properties, while Epalrestat remains approved only in a few countries for diabetic neuropathy [61]. Nevertheless, renewed interest is emerging, as the role of AKR1B1 in cancer, fibrosis, and oxidative stress becomes clearer, inspiring the development of next-generation, tissue-specific inhibitors.

## 6. Future Directions

Looking ahead, future research should leverage cutting-edge technologies to unravel the multifaceted role of AKR1B1 in EMT and disease progression. Single-cell transcriptomics and spatial proteomics could provide unprecedented resolution in mapping AKR1B1 expression and activity across different cell populations and tissue microenvironments, clarifying its cell-type-specific contributions to fibrosis and tumor heterogeneity. Moreover, the development of in silico models based on predictive algorithms for drug screening and structure-guided design may accelerate the identification of selective and bioavailable inhibitors. These innovative strategies could transform AKR1B1 from a mechanistic biomarker into a viable therapeutic target, opening new avenues for intervention in EMT-driven diseases.

From a future perspective, an especially relevant aspect is the role of AKR1B1 in chemoresistance. EMT is a well-recognized contributor to drug resistance [62], and AKR1B1 has recently been observed to be overexpressed in chemoresistant cells [63], suggesting that it may contribute to therapy failure by promoting EMT. Future studies should aim to determine whether AKR1B1 directly mediates chemoresistance through EMT-related pathways and whether pharmacological inhibition of AKR1B1 can restore drug sensitivity.

Finally, while EMT is also a physiological process essential for embryogenesis—driving events such as gastrulation, neural crest migration, and organogenesis—the role of AKR1B1 in developmental EMT remains entirely unexplored. Clarifying whether AKR1B1 contributes to embryonic development may not only broaden our understanding of its biological functions but also provide insights into potential links with developmental abnormalities.

## Data Availability

No new data were created or analyzed in this study. Data sharing is not applicable to this article.
